# Proteotoxic stress targeted therapy (PSTT): induction of protein misfolding enhances the antitumor effect of the proteasome inhibitor bortezomib

**DOI:** 10.18632/oncotarget.246

**Published:** 2011-03-27

**Authors:** Nickolay Neznanov, Andrei P. Komarov, Lubov Neznanova, Patricia Stanhope-Baker, Andrei V. Gudkov

**Affiliations:** ^1^ Department of Cell Stress Biology, Roswell Park Cancer Institute, Buffalo, NY 14263; ^2^ Cellecta, Inc. Mountain View, CA 94043; ^3^ Cleveland BioLabs, Inc., Buffalo, NY 14263

**Keywords:** bortezomib, puromycin, hyperthermia, heat shock, apoptosis, cancer treatment, translation, ubiquitination, protein degradation, protein denaturing

## Abstract

Proteotoxic stress (PS) is generated in cells under a variety of conditions involving accumulation of misfolded proteins. To avoid the toxicity of unmitigated PS, cells activate the heat shock response (HSR). HSR involves upregulation of factors such as ubiquitin and the non-housekeeping chaperone Hsp70 which assist with metabolism of aberrant proteins. The PS-HSR axis is a potential anticancer treatment target since many tumor cells display constitutive PS and dependence on HSR due to their rapid rates of proliferation and translation. In fact, induction of PS via stimulation of protein misfolding (hyperthermia), inhibition of proteasomes (bortezomib) or inhibition of Hsp90 (geldanamycin) have all been considered or used for cancer treatment. We found that combination of bortezomib with an inducer of protein misfolding (hyperthermia or puromycin) resulted in enhanced PS. HSR was also induced, but could not mitigate the elevated PS and the cells died via largely p53-independent apoptosis. Thus, combination treatments were more cytotoxic in vitro than the component single treatments. Consistent with this, combination of non-toxic doses of puromycin with bortezomib significantly increased the antitumor activity of bortezomib in a mouse model of multiple myeloma. These results provide support for using combination treatments that disrupt the balance of PS and HSR to increase the therapeutic index of anticancer therapies.

## INTRODUCTION

Proteotoxic stress (PS) is generated by accumulation of misfolded proteins in cells under a variety of conditions including hyperthermia, hypoxia, and exposure to denaturing agents or drugs that inhibit proteasome or chaperone activities [1-3]. PS can be toxic if misfolded proteins accumulate and aggregate in the cells. To avoid the toxicity of PS, cells activate an adaptive response, known as the heat shock response (HSR)[4].

The adaptive HSR involves induction of factors and pathways that serve to mitigate the burden of misfolded proteins. Thus, additional chaperones (e.g., Hsp70) are synthesized to assist in metabolism of misfolded proteins, ubiquitin is upregulated to allow for more proteasome-mediated degradation, and general translation is attenuated [5,6]. In mammalian cells, the adaptive HSR depends upon activation of the transcription factor HSF1 [7]. HSF1 is required for expression of the inducible form of Hsp70 as well as other chaperones and some stress-related transcription factors [1].

Many tumors and tumor-derived cells contain elevated levels of misfolded proteins due to the high level of translation associated with their rapid rate of proliferation, as well as specific intracellular conditions, such as glycolysis-related acidification [8,9]. Therefore, these tumor cells exist under conditions of constant PS and exhibit constitutive activation of HSR. The elevated expression in tumors of both “housekeeping” heat shock proteins (HSP) such as the chaperone Hsp90 and the inducible form of Hsp70 is a reflection of their level of PS. Thus, the ability to properly respond to PS is especially important for survival of tumor cells. This is illustrated by the finding that genetic or pharmacological inhibition of Hsp70 synthesis in response to PS in tumor cells increased the toxicity of PS and resulted in apoptosis [10-12]. In addition, HSF1 knock-out mice showed reduced frequency of tumor formation in response to oncogene expression or exposure to oncogenic chemicals as compared to wild type mice [13-14]. Thus, HSF1-related gene expression represents a principal mechanism by which cancer cells survive in the face of constant PS.

Since tumor cells are particularly prone to PS and, thereby, dependent upon effective HSR, targeting of HSR has been recognized as an attractive strategy for anti-cancer therapy. Previous studies have demonstrated that it is possible to manipulate the toxicity of PS induced by different stimuli, such as hyperthermia, proteasome inhibitors and chaperone inhibitors to achieve greater tumor cell killing. One way to increase the toxicity of PS is to suppress the adaptive HSR by blocking Hsp70 synthesis. For example, we showed that the anti-malaria drug quinacrine and its close analogue 9-amino-acridine prevented PS-specific induction of Hsp70 and increased the toxicity of PS for both tumor cells in vitro and tumors *in vivo* [12]. Other studies demonstrated increased cytotoxicity through combination of the Hsp90 inhibitor geldanamycin with cisplatin (an inhibitor of HSF1 DNA binding) [15] or combination of hyperthermia with siRNA-mediated knockdown of Hsp70 [10]. In these three studies, the first component of the combination treatment is a PS inducer, while the latter component acts as an inhibitor of HSR.

We proposed an alternative approach for increasing the toxicity of PS in tumor cells based on the idea that the HSR capacity of a cell is limited by the expression levels of a small number of genes (e.g., those encoding inducible chaperones, such as Hsp70 [3,7], and ubiquitin [16]). The roles of chaperones and ubiquitin in protein metabolism that lead to effective HSR require that they are available in sufficient amounts to interact with most of the misfolded protein molecules present in the cell [2]. Thus, although HSR is highly inducible by PS and typically very effective, we hypothesized that it might be possible to increase the level of misfolded proteins such that it cannot be matched by the cell's HSR (capacity to synthesize chaperones and ubiquitin). Such “enhanced proteotoxic stress” (EPS) would be expected to be highly cytotoxic, especially for tumor cells which are constitutively coping with pre-existing PS. In this study, we show that EPS can be generated through combination of a treatment that causes misfolding of proteins (hyperthermia or puromycin) with the proteasome inhibitor bortezomib. We demonstrate that simultaneous induction of protein misfolding and inhibition of proteasome-mediated degradation induces EPS in several different types of tumor cells and leads to cell death in vitro and suppression of tumor growth in vivo. The tested drug combinations show greater efficacy than either single component treatment, both in inducing PS (generating EPS) and in killing tumor cells in vitro and in vivo. These results indicate that such combinatorial PS-targeting therapies hold substantial promise as improved cancer treatments.

## RESULTS

### Induction of EPS by a combination of hyperthermia and bortezomib

**Figure 1:**
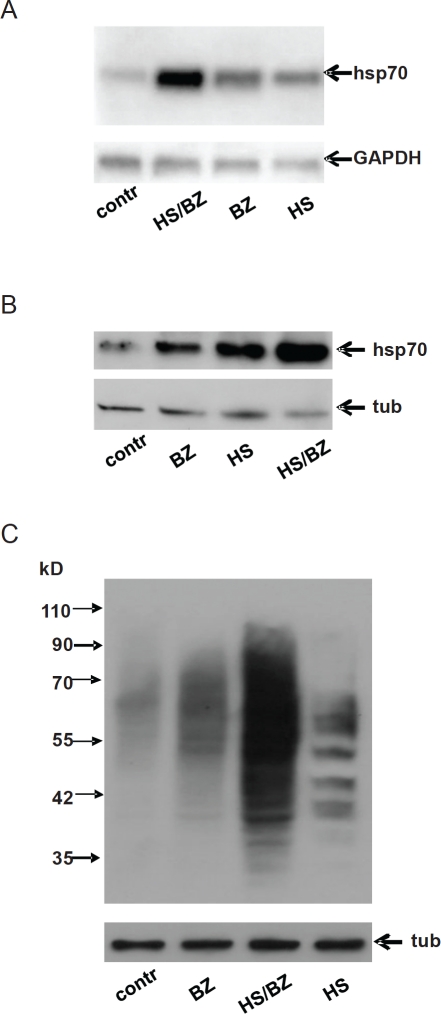
Combined treatment with hyperthermia and bortezomib produces enhanced proteotoxic stress (EPS) **A.** Northern blot analysis of HT1080 cells left untreated (contr) or treated with 100nM bortezomib for 5 h (BZ), hyperthermia (heat shock at 43°C for 1 h, HS), or hyperthermia together with 100nM bortezomib (HS/BZ). For the combined treatment, bortezomib was added just before heat shock (43°C for 1 h), after which the cells were transferred to 37°C and incubated for an additional 4 h. 10μg of total RNA from each sample was analyzed by Northern hybridization with the Hsp70A1probe and the GAPDH probe (as a loading control). **B.** Western blot analysis of protein extracts (10μg) from HT1080 cells treated as described in panel A. Blots were probed with anti-Hsp70 antibody and anti-tubulin antibody (tub) as a loading control. **C.** Total protein ubiquitination in HT1080 cells treated as described in panel A. Western blotting was performed using 10 μg of protein extract. Blots were probed with anti-ubiquitin antibody (top panel) and anti-tubulin antibody (bottom panel, tub) as a protein loading control.

The objective of this study was to test whether we could create EPS that would be cytotoxic in tumor cells without direct inhibition of HSR. We hypothesized that the severity of PS would be enhanced by combining a treatment that induces protein misfolding, such as hyperthermia [17], with a treatment that prevents degradation of misfolded proteins, such as the proteasome inhibitor bortezomib [3]. Using inducible Hsp70 expression as a marker of PS, we found that combined treatment with hyperthermia and bortezomib resulted in a much higher level of PS than either agent alone (Fig. 1A, B). As expected based on the well-established relationship between Hsp70 expression and PS, we also observed much higher levels of ubiquitinated proteins (presumably misfolded) in cells treated with hyperthermia and bortezomib than in cells treated with either agent alone (Fig. 1C). These data indicate that combined treatment with hyperthermia and bortezomib produces EPS.

**Figure 2:**
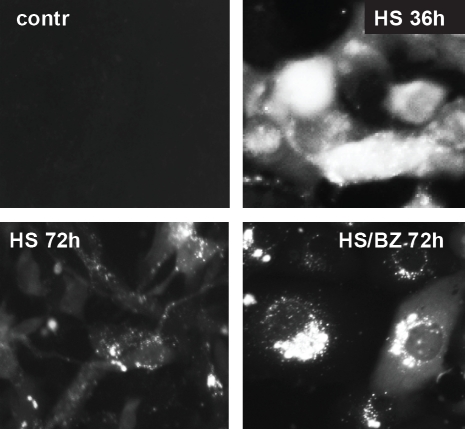
Accumulation of heat shock-induced GFP in aggresomes in bortezomib treated cells H1299-HSE/GFP cells were left untreated (contr) or treated with hyperthermia (HS) at 43°C for 1 h to induce HSF1-dependent GFP synthesis. After heat shock, the cells were transferred to 37°C and incubated for the indicated amounts of time (36 or 72 h) with or without 40nM bortezomib (BZ). The synthesis (HS36h), degradation (HS72h), and accumulation in aggresomes (HS/BZ72h) of GFP were monitored by fluorescent microscopy.

The EPS generated by hyperthermia+bortezomib was not effectively mitigated by coincident induction of HSR in co-treated cells, as illustrated by aggregation of misfolded proteins in the cells (Fig. 2). Heat shock treatment (1 hour at 43°C) of H1299 cells carrying an HSF1-regulated GFP expression construct (H1299-HSE/GFP) resulted in a high level of GFP expression which was still evident 36 hours later (Fig. 2B). By 72 hours after heat shock, most of the GFP protein was degraded in cells that did not receive any further treatment (Fig. 2C, 2Ca), indicating effective HSR. In contrast, if the heat-shocked H1299-HSE/GFP cells were incubated in the presence of bortezomib for the subsequent 72 hours, GFP accumulated in aggresomes (Fig. 2D, 2Da). These data suggest that the combination of an inducer of protein misfolding (hyperthermia) with an inhibitor of proteasomal degradation (bortezomib) produces a level of PS that the cell cannot mitigate, even though HSR is induced by the treatments as well.

**Figure 3:**
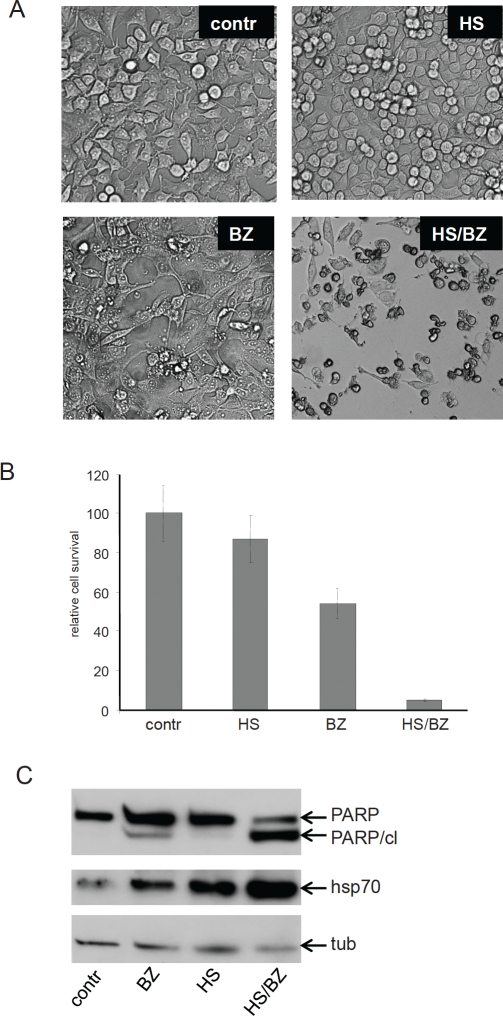
EPS generated by combined hyperthermia and bortezomib is more cytotoxic than either treatment alone **A. Combination of hyperthermia and bortezomib is toxic to HeLa cells.** HeLa cells were left untreated (Contr) or treated with hyperthermia (43°C heat shock for 1h) (HS), 40 nM bortezomib for 18h (BZ), or their combination (HS/BZ). **B.** Methylene-blue-based quantitation of the results of the cell survival assay presented in panel A. **C. Combination of hyperthermia and bortezomib induces apoptosis in HeLa cells.** Total protein extracts from HeLa cells treated as described in (A) were analyzed by Western blotting with anti-PARP, anti-Hsp70, and anti-tubulin (tub, control for protein loading) antibodies. Full-length PARP and the apoptosis-specific cleaved PARP fragment (PARP cl.) are indicated. Hsp70 was assessed as a marker of adaptive HSR.

We next determined whether EPS generated by treatment with hyperthermia+bortezomib resulted in enhanced cytotoxicity. Under conditions of standard PS, such as that generated by bortezomib or hyperthermia alone, the induced adaptive HSR is usually sufficient to allow cell survival. For example, HT1080, HCT116 (data not shown) and HeLa (Fig. 3A, B) cells can completely recover from 1 hour incubation at 43°C during additional incubation at 37oC. Treatment with 40 nM of bortezomib for 18 hours induces a similar level of PS (data not shown), but was slightly more toxic to HeLa cells than hyperthermia (Fig. 3A, B). This is likely due to the fact that proteasome inhibition has other effects in addition to induction of PS, such as causing accumulation of the pro-apoptotic factor p53 and suppressing the pro-survival activities of NF-kappaB and Akt [18.^19^]. Nevertheless, both of the above-described PS-inducing treatments (heat shock and bortezomib) leave the majority of treated cells alive. In contrast, we found that combination of the two treatments resulted in a much higher level of cell death (Fig. 3A, B). Analysis of PARP cleavage demonstrated that cell death induced by hyperthermia+bortezomib is accompanied by activation of caspases indicative of apoptosis (Fig. 3C). These data demonstrate that the extent of cell death in cells treated with hyperthermia+bortezomib correlated with the increased level of PS generated by the combination treatment (Fig. 1). This supports our hypothesis that the cytotoxity of the combination treatment is due to accumulation of misfolded proteins to a level that exceeds the capacity of the cell's natural adaptive HSR.

### Induction of EPS by a combination of puromycin and bortezomib

**Figure 4:**
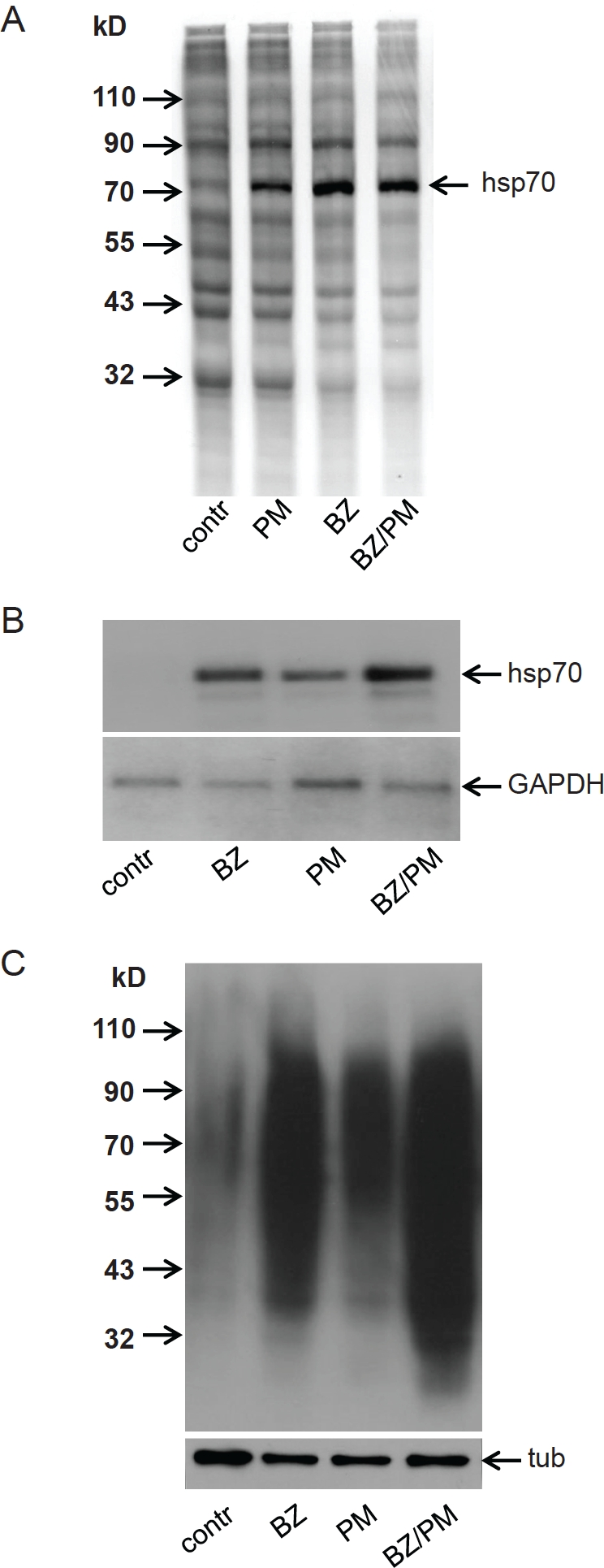
Combined treatment with bortezomib and puromycin produces enhanced proteotoxic stress conditions (EPS) **A. Puromycin induces PS and does not affect protein synthesis.** HT1080 cells were treated for 4 h with puromycin (1 μM) (PM), bortezomib 100 nM (BZ), or their combination (PM/BZ), and then labeled with S^35^—methionine for 60 min. S^35^-labeled proteins from HT1080 cells were analyzed by SDS-PAGE and autoradiography. Protein extracts from equal numbers of cells were loaded on the gel. The position of Hsp70 (an indicator of PS) is marked by the arrow. **B. Combined treatment with bortezomib and puromycin induces EPS.** Northern blot analysis was performed using RNA from HT1080 cells treated for 18 h with 40 nM bortezomib (BZ), 400 nM puromycin (PM), or their combination (BZ/PM). Blots were hybridized with probes for Hsp70 (top panel) and GAPDH (RNA loading control, bottom panel). **C. Total protein ubiquitination in response to bortezomib, puromycin and their combination.** Western blotting was performed using 10 μg of total protein extract from HT1080 cells treated as described in (B). Blots were probed with anti-ubiquitin antibody (top panel) and anti-tubulin antibody (bottom panel, tub) as a protein loading control.

**Figure 5:**
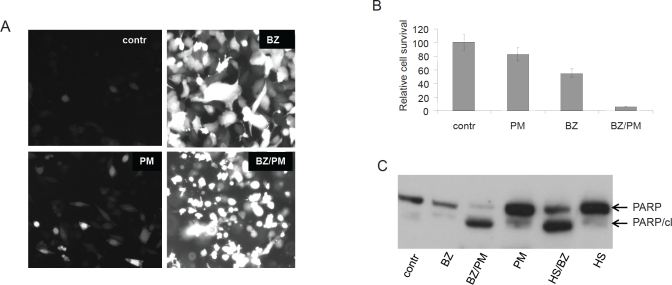
Combined treatment with bortezomib+puromycin is more cytotoxic than treatment with either drug alone **A. Toxic effect of EPS induced by combination of bortezomib and puromycin.** GFP expression in HeLa-HSE/GFP cells treated with 40 nM bortezomib (BZ), 400 nM puromycin (PM), or their combination (BZ/PM) for 18h. Toxicity is indicated by changes in the cells' morphology and their detachment from the plate. **B.** Cell survival assay with HeLa-HSE/GFP cells treated as described in (A). Methylene blue was used for quantitation. **C. Apoptosis is induced in HT1080 cells in response to EPS.** Total protein extracts from HT1080 cells treated with bortezomib (BZ), puromycin (PM), hyperthermia (HS.), and their combinations as described in (A) and in the legend to Figure 3 were analyzed by Western blotting with anti-PARP antibody. Full-length PARP and the apoptosis-specific cleaved PARP fragment (PARP/cl) are indicated by arrows. Equivalent amounts of protein were loaded in all lanes.

Like hyperthermia, some pharmacological agents also induce proteins' misfolding. For example, the antibiotic puromycin, which acts as a non-functional analogue of aminoacyl tRNA, causes premature termination of translation and accumulation of aborted, improperly folded translation products [20]. We therefore hypothesized that puromycin, similar to hyperthermia, would enhance the antitumor effect of bortezomib. As shown in Figure 4, treatment of HT1080 tumor cells in vitro with a combination of puromycin and bortezomib caused greater PS and adaptive HSR than treatment with either drug alone. The classic marker of adaptive HSR, Hsp70, was upregulated to a greater extent at both the protein (Fig. 4A) and mRNA (Fig. 4B) levels by the drug combination as compared to either single drug. Notably, induction of HSR (as judged by induction of Hsp70 expression) by puromycin was observed at a concentration that does not cause suppression of general translation (Fig. 4A). Similar effects of bortezomib, puromycin and their combination on induction of HSR were observed in HeLa cells carrying an Hsp70-regulated GFP expression constructed (HSE/GFP) (Fig. 5A). Consistent with the effects of puromycin+bortezomib on Hsp70, we found that the combination treatment also led to greater accumulation of ubiquitinated proteins in HT1080 cells as compared to treatment with either drug alone (Fig. 4C). Taken together, these results indicate that combination of bortezomib with puromycin (as with hyperthermia) generates EPS within the treated cells.

Generation of EPS by combined puromycin+bortezomib treatment correlated with synergistic cytotoxicity of the two drugs. Treatment of HeLa HSE/GFP cells with 400 ng/ml puromycin and 40 nM bortezomib for 18 hours resulted in significantly reduced cell viability as compared to treatment with either drug alone using the same dose/time (Fig. 5A, B). The observed reduction in cell viability correlated with appearance of morphological (small, rounded, detached cells (Fig.5A)) and biochemical (Fig. 5C) signs of apoptosis. These data support our hypothesis that combination treatments resulting in accumulation of misfolded proteins to levels that exceed the salvaging capacity of HSR will lead to cell death and demonstrate that such death proceeds, at least in part, via apoptosis.

### Involvement of p53 in the response of cells to EPS

**Figure 6:**
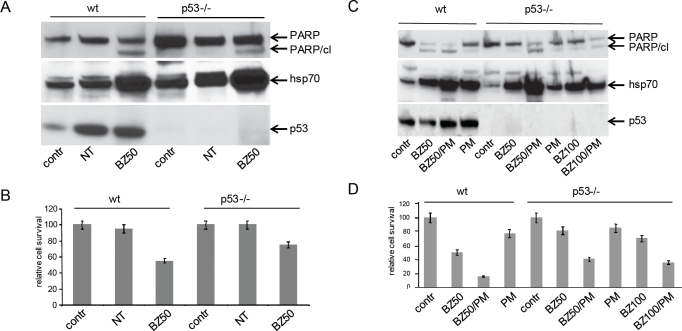
Role of p53 in cytoxicity induced by PS and EPS **A. Stabilization of p53 by nutlin-3a is not toxic for wt HCT116 cells and p53 is not required for PS-induced toxicity.** P53 wild type (wt) and p53-/- HCT116 (p53-/-) cells were left untreated (contr) or treated with nutlin-3a (NT, 100 μM) or bortezomib (50 nM, BZ50) for 24 h. Total protein extracts (10 μg) were analyzed by Western blotting with anti-PARP (top panel), anti-Hsp70 (middle panel) and anti-p53 (bottom panel) antibodies. The anti-PARP antibody detects both full-length PARP and the apoptosis-specific cleaved fragment (PARP/cl). **B.** Methylene blue-based quantitation of cell survival assay for the cells described in (**A**). **C. The extent of PS/EPS-induced apoptosis is greater in wild type cells than in p53-/- cells.** P53 wild type HCT116 cells (wt) and HCT116p53-/- cells (p53-/-) were left untreated (contr) or treated with bortezomib (BZ, 50 or 100 nM), puromycin (PM, 400 ng/ml) or their combination as indicated below each lane for 24 h. Western blotting was performed as described for (**A**). Equivalent amounts of protein were loaded in all lines. **D.** Methylene blue-based quantitation of cell survival assay for the cells described in(**C**). Results are the average of two experiments as described in Materials and Methods.

Inhibition of proteasome activity results in stabilization and accumulation of the key pro-apoptotic protein p53 [21]. To examine the involvement of p53 in EPS-induced cytotoxicity, we used isogenic p53-/- and p53 wild type HCT116 cell lines [22] and compared the effects of nutlin-3a, an MDM2 inhibitor that specifically prevents p53 degradation [23,24], with those of bortezomib. Both nutlin-3a and bortezomib stabilize p53 presumably by preventing its degradation by proteasomes [24,25]; however, only bortezomib also induced PS/HSR. These effects are illustrated in Figure 6A for HCT116 cells with wild type p53, using Hsp70 as a marker of PS/HSR. Under the conditions used in this experiment, stabilization of p53 by nutlin-3a was not sufficient to induce apoptosis. This was illustrated by a lack of PARP cleavage (Fig. 6A) and no change in cell survival (Fig. 6B). In contrast, bortezomib treatment (50 nM for 24 hours) producing the same level of p53 stabilization caused substantial apoptosis of p53 wild type cells (as indicated by PARP cleavage and cell survival assays). These results indicate that the toxicity of bortezomib is not due to stabilization of p53, but is correlated with induction of PS. Analysis of p53-/- HCT116 cells provided additional support for this conclusion. Thus, even in the complete absence of p53, bortezomib (but not nutlin-3a) induced both Hsp70 expression indicative of PS/HSR and noticeable PARP cleavage indicative of apoptosis (Fig. 6A). The cytotoxicity of bortezomib, but not nutlin-3a, towards p53-/- cells was also evident in cell survival assays (Fig. 6B).

The experiments described above (Fig. 6A, B) demonstrate that the accumulation of p53 induced by nutlin-3a is not sufficient on its own to trigger apoptosis in HCT116 cells and that p53 is not required for PS-induced cytotoxicity. Nevertheless, p53 activity might play a role in the efficiency of apoptosis induced by PS or EPS. This possibility is supported by the greater level of PARP cleavage and cell death seen in bortezomib-treated p53 wild type HCT116 cells as compared to similarly treated p53-/- HCT116 cells (Fig. 6A, B) To further evaluate the role of p53 in the toxicity of PS, we treated p53 wild type and p53-/- HCT116 cells with 50 nM bortezomib, 400 ng/ml puromycin or their combination for 24 hours and evaluated induction of PS/HSR (by Hsp70 expression assay) and apoptosis (by PARP cleavage and cell survival assays).

In both cell lines, treatment with either 50 nM bortezomib or 400 ng/ml puromycin resulted in PS and treatment with their combination produced EPS (as evidenced by the level of Hsp70 induction, Fig.6C). PS resulting from treatment with puromycin alone was a weak inducer of apoptosis in p53 wild type and p53-/- HCT116 cells (illustrated by the low level of PARP cleavage (Fig. 6C) and cell death (Fig. 6D)). In contrast, while PS following from treatment with bortezomib alone did lead to noticeable apoptosis in p53-/- HCT116 cells, the extent of apoptosis was greater in similarly treated p53 wild type cells (Fig. 6C, D). These data are consistent with those shown in Figure 6B for similar treatment of p53-positive and —negative HCT116 cells with bortezomib alone. The extent of apoptosis induced by treatment of p53-/- cells with bortezomib alone was not substantially enhanced even when the dose of drug was doubled to 100 nM (Fig. 6D).

Unlike treatment with either drug alone, combined treatment with puromycin+bortezomib resulted in substantial apoptosis in p53 wild type and p53-/- HCT116 cells. The extent of apoptosis in combination-treated p53-/- cells was not dependent upon bortezomib dose (50 nM versus 100 nM). Even in p53 wild type cells, which showed an apoptotic response to bortezomib alone, the response was stronger following combination treatment. As for treatment with bortezomib alone, the extent of apoptosis induced by bortezomib+puromycin was greater in the p53 wild type cells than in the p53-/- cells. Taken together, the data shown in Fig. 6A-D indicate that PS/EPS-induced apoptosis is largely p53-independent: p53 activity is not required for PS/EPS-induced apoptosis; however, it does appear to contribute to the extent of the observed apoptosis.

### Anti-tumor efficacy of PSTT in a mouse model of multiple myeloma

**Figure 7:**
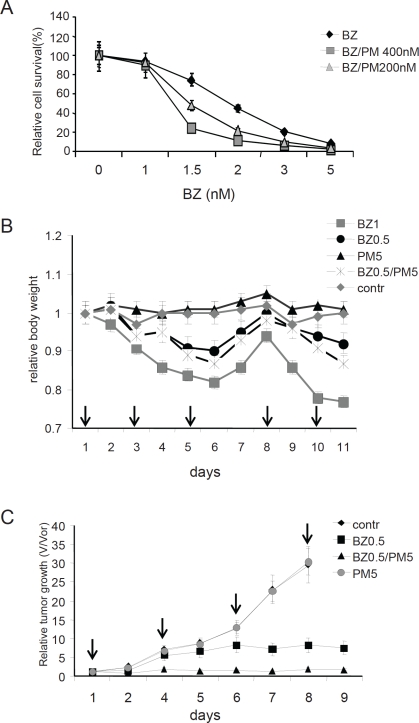
Effect of EPS on MPC-11 multiple myeloma tumor growth **A. Combinatorial toxicity of bortezomib and puromycin on MPC-11 multiple myeloma cells in vitro.** MPC-11 cells growing in culture were treated with the indicated amounts (x-axis) of bortezomib alone or in combination with 200 nM or 400 nM puromycin for 18 h. Cell survival was estimated by counting trypan blue-excluding cells. The data shown represent the average of two independent experiments. **B. *in vivo* drug toxicity.** General toxicity of treatment regimens was evaluated by daily measurement of mouse body weight. Groups of 3 BALB/c mice were injected with the indicated doses of drugs on the days marked by arrows. Each data point represents the average weight in the group relative to the average weight in the group before first drug injection. **C. Effect of combined bortezomib + puromycin treatment on MPC-11 multiple myeloma tumor growth in vivo.** Groups of 5 BALB/c mice carrying syngeneic transplanted MPC-11-derived tumors (see Materials and Methods) were injected (i.p.) with PBS (contr), 0.5 mg/kg bortezomib (BZ0.5), 5 mg/kg puromycin (PM5), or 0.5 mg/kg bortezomib + 5 mg/kg puromycin (BZ0.5/PM5) on the days marked with arrows. Tumors were measured daily. The graph shows the average relative tumor growth within each treatment group (relative tumor growth=tumor volume on day of measurement (V) divided by tumor volume of the day of the first drug injection (Vor). Error bars indicate standard deviation. Animals were sacrificed if their tumors reached 1200 mm^3^ in volume.

Although bortezomib is FDA-approved for treatment of multiple myeloma, its clinical use is complicated by high general toxicity [26,27]. Based upon our in vitro data showing that combining puromycin with bortezomib increased tumor cell killing (see above), we hypothesized that combined administration of puromycin with bortezomib *in vivo* would allow an anti-tumor therapeutic effect to be reached at lower doses of bortezomib, thereby reducing general toxicity. To test this possibility, we used a model consisting of MPC11 mouse multiple myeloma cells grown as syngeneic tumors in Balb/c mice. Before performing the *in vivo* study, we tested the effect of different concentrations of bortezomib (1, 1.5, 2, 3 or 5 nM) alone or in combination with different concentrations of puromycin (200 or 400 ng/ml) on survival of MPC11 cells growing in culture (Fig. 7A). As expected, increasing concentrations of bortezomib resulted in increased cell death. In the middle part of the dose-response curve (1.5 and 2 nM bortezomib), increased cytotoxicity was observed with inclusion of puromycin in the cell treatment. This effect of puromycin was also dose-dependent, being stronger with 400nM than with 200nM puromycin. These data indicate that, as for HeLa and HT1080 cells (see above), combination of puromycin and bortezomib results in enhanced death of MPC11 mouse MM cells.

We next evaluated whether combination of puromycin and bortezomib would also lead to killing of MPC11 cells growing as solid tumors in mice. Four groups of BALB/c mice (5 animals/group) were injected sub-cutaneously on both sides of their bellies with 2x10^5^MPC11 cells. Mice were given their first drug injection (intraperitonealy) when at least one tumor reached 50 mm^3^ in size. Groups were injected with PBS (negative control), 0.5 mg/kg bortezomib alone, 5 mg/kg puromycin alone, or 0.5 mg/kg bortezomib + 5 mg/kg puromycin. We chose to use the 0.5 mg/kg dose of bortezomib since the reported effective antitumor dose of bortezomib (1 mg/kg dose [28]) caused significant general toxicity as indicated by mouse weight loss (Fig. 7B) and combination of 1 mg/kg bortezomib with 5 mg/kg puromycin had the same anti-tumor effect and same toxicity as 1 mg/kg bortezomib used alone (data not shown). Reduction of the bortezomib dose to 0.5 mg/kg resulted in much lower general toxicity, but also led to a significant decrease in its anti-tumor effect (Fig. 7B and data not shown). Importantly, however, when this lower dose of bortezomib (0.5 mg/kg) was combined with 5 mg/kg puromycin (itself non-toxic and ineffective as a single agent), inhibition of tumor growth was enhanced without a substantial increase in general toxicity (Fig. 7B, C). The tested combination of 0.5 mg/kg bortezomib + 5 mg/kg puromycin was as effective in suppressing tumor growth as the therapeutically optimal bortezomib dose (1.0 mg/kg) while showing less severe toxicity, as judged by mouse body weight loss (Fig.7B, C, data not shown).

## DISCUSSION

The maintenance of protein homeostasis in cells requires the activities of chaperones, such as Hsp90, Hsp70, and Hsp27, and the ubiquitin-proteasome system [29], which together serve to inactivate and degrade misfolded proteins. Elevation of the intracellular concentration of misfolded proteins can result from hyperthermia (heat shock), increased translation, exposure to protein-denaturing agents, or treatment with agents that interfere with natural mechanisms of misfolded protein processing, such as the Hsp90 inhibitor geldanamycin or the proteasome inhibitor bortezomib [15,30-32]. Regardless of the underlying cause, accumulation of misfolded proteins leads to PS, which, if not mitigated, can lead to protein aggregation and cell death, often via apoptosis [33,34]. Cells are typically able to survive PS, though, due to activation of the adaptive HSR, which involves induction of HSF1-mediated transcription of genes encoding additional chaperones, such as Hsp70, and other stress-related proteins [2,31].

Effective HSR is particularly important for survival of cancer cells, which are characterized by constitutively high levels of PS and coincident dependence on HSF1 activity [13,35]. This presents the attractive possibility of targeting PS and HSR as anti-cancer therapies and indeed, inducers of PS, such as chaperone inhibitors and proteasome inhibitors (e.g., bortezomib) have been introduced as such. It is likely, however, that the effectiveness of these drugs is limited by the cell's natural HSR. Therefore, strategies aimed at increasing the toxicity of PS towards tumor cells, such as suppression of HSR, have been explored [10,12,13,^15^]. For example, in our previous publication, we described the ability of the anti-malaria drug quinacrine to block expression of Hsp70 in response to PS [12]. Due to its effect on Hsp70, combination of quinacrine with inducers of PS, such as hyperthermia, bortezomib, or geldanamycin resulted in increased cytotoxicity in vitro and increased suppression of tumor growth *in vivo* in syngeneic transplant mouse models [12]. These findings, together with those from a number of other studies, demonstrate that suppression of HSR under conditions of PS increases the toxicity of PS, induces cancer cell death, and affects tumor growth [13].

In the current study, we tested an alternative approach to increasing the anti-tumor toxicity of PS inducers. Rather than blocking HSR in response to a given level of PS, this alternative approach leaves HSR unchanged while increasing the level of PS. We hypothesized that combining PS inducers with different target pathways (e.g., combining a treatment that causes misfolding of proteins with a treatment that prevents their degradation) would create EPS that would exceed the capacities of the cell's adaptive HSR and prove intolerable for cell survival. Such a strategy might be expected to be particularly effective for killing of tumor cells since their HSR capacity is partially exhausted due to pre-existing PS.

Our results demonstrate that combined treatment with the proteasome inhibitor, bortezomib, and an inducer of protein misfolding (either hyperthermia or puromycin) produced EPS and that EPS correlated with cytotoxicity. Thus, in several cell lines in vitro, both combination treatments greatly increased the level of PS (as evidenced by the level of Hsp70 expression and accumulation of ubiquitinated proteins) over that induced by either single component of the treatment used alone. Similarly, for both hyperthermia+bortezomib and puromycin+bortezomib, the combination treatment was much more cytotoxic than single treatments. The enhanced cell killing achieved with the combination treatments does not rely on induction of any toxic process other than the accumulation of misfolded proteins caused by bortezomib alone, but simply increases formation of misfolded proteins such that the PS is “enhanced.”

Proteasome inhibition leads to stabilization and accumulation of p53, a major pro-apoptotic regulator of cell death/survival [36-38]. This plays an important role in the anti-tumor efficacy of bortezomib [25,39]. Accordingly, the resistance of some tumors to proteasome inhibitors has been attributed to their p53-negative status or failure of the proteasome inhibitor to stabilize p53 [40-42]. Moreover, the expression of wild type p53 in B lymphomas made them sensitive to bortezomib in vitro and *in vivo* [35].In other studies, however, the accumulation of p53 and the expression of wild type p53 in p53-/- tumor cells did not change the sensitivity of cells to apoptosis induced by proteasome inhibitors [43,44]. We found that combination treatments that produced EPS in cultured tumor cells also reduced cell survival and induced PARP cleavage, indicative of apoptosis. Given the known effects of bortezomib on p53, we performed experiments to evaluate the role of p53 in EPS-induced cell death. Use of nutlin-3a, a compound that specifically stabilizes p53 without inducing by PS, showed that stabilization of p53 is not, in itself, sufficient to trigger apoptosis in HCT116 cells. Comparison of p53 wild type and p53-/- HCT116 cells treated with bortezomib, puromycin, or their combination revealed that: (i) p53 is not absolutely required for induction of apoptosis by PS or EPS; and (ii) p53 does, nevertheless, contribute to the extent of PS/EPS-induced apoptosis. The first conclusion ((i) above) was drawn from our data showing that treatment with bortezomib alone, and to a much greater extent, with bortezomib+puromycin, resulted in cleavage of PARP and reduction of cell survival in p53-deficient, as well as p53 wild type, HCT116 cells. Despite the accepted involvement of p53 in the mechanism of action of bortezomib [25], there are previous publications that describe killing of p53-/- cells by proteasome inhibitors [43-45] which support our finding that PS/EPS-induced apoptosis is, at least in part, p53-independent. The second conclusion ((ii) above) is supported by our finding that p53-/- cells were less sensitive to bortezomib or bortezomib+puromycin than p53 wild type cells were. Thus, PARP cleavage and cell death were observed in p53-deficient cells exposed to PS- or EPS-inducing treatments, but were observed at higher levels in similarly treated p53 wild type cells. Our findings that EPS is more cytotoxic than PS and that EPS-induced apoptosis is at least partially p53-independent suggest that EPS may be a promising treatment for p53-negative tumors in vivo. The enhanced cytotoxicity afforded by puromycin+bortezomib treatment might be particularly important *in vivo* since the time of bortezomib activity is limited *in vivo* by its detoxification in the liver by oxidative deboronation [46].

Bortezomib is an FDA-approved drug for treatment of multiple myeloma. Although multiple myeloma cells are highly sensitive to bortezomib in vitro and die within 18 hours of exposure to just 5nM bortezomib, multiple myeloma tumors growing in mice are rather resistant to bortezomib treatment. In syngeneic and xenograft mouse multiple myeloma models, non toxic doses of bortezomib did not completely eliminate tumors, but only delayed their growth [28,47]. Bortezomib has shown efficacy in human multiple myeloma patients [48]; however, its application is limited by induction of non-tumor-specific toxicity. The typical dose of bortezomib used in the clinic is 1-1.3 mg/m2. The drug is administered 4 times with 3 days intervals during the twice weekly treatment's cycle [38] or 4 times up to 1.6 mg/m2 during the weekly treatment cycle [49]. The most frequent complication of bortezomib treatment is development of peripheral neuropathy, which is detected in 40-64% of treated patients with 14-30% requiring reduction of the drug dose or discontinuation of treatment [50,51].

Our findings in cultured cells demonstrated that tumor cell killing could be increased by combining bortezomib with puromycin. Therefore, we analyzed the effect of bortezomib+puromycin combination treatment as compared to single agent treatment on growth of MPC11 MM syngeneic tumor transplants in BALB/c mice. Under the treatment regimens used, high dose (1 mg/kg) bortezomib significantly delayed tumor growth, but also caused substantial general toxicity (as evidenced by mouse weight loss). 5 mg/kg puromycin was non-toxic and did not have any effect on tumor growth when administered alone and did not increase the anti-tumor toxicity of 1 mg/kg bortezomib treatment (data not shown). However, with a lower dose of bortezomib (0.5 mg/kg) that showed low general toxicity, co-treatment with 5 mg/kg puromycin significantly reduced tumor growth as compared to low dose bortezomib alone. This combination of drugs produced low general toxicity. The ability to achieve tumor suppression with a reduced dose of bortezomib through its combination with puromycin is an attractive approach for anti-cancer therapy since it allows targeting of PS without the general toxicity that is associated with high doses of bortezomib. Overall, our data support the anti-cancer therapeutic potential of PSTT involving combinations of proteasome inhibitors and other drugs that target the PS-HSR axis, such as puromycin or quinacrine [12].

## MATERIALS AND METHODS

### Cell culture, lentiviral vectors, and drugs

HT1080, HeLa, H1299, and HCT116 cells were cultured in Dulbecco modified Eagle's medium (Invitrogen/Gibco BRL, Carlsbad, CA) supplemented with 10% fetal calf serum. MPC11 cells were grown in complete RPMI-1640 medium supplemented with 10% fetal calf serum, 2 mM L-glutamine, 100 g/ml streptomycin, 100 U/ml penicillin, 50 μg/ml Gentamicin (Life Technologies, Grand Island, NY). The construct for HSF1-specific EGFP expression (“HSE-GFP”) was generated by inserting 6 copies of an HSF1 regulatory element (HSE; 5'-CAGAACGTTCTAG-3') [52] upstream of the minimal CMV promoter in the PTR-mCMV-EGFP lentiviral vector (Cellecta, Inc. Mountain View, CA). Cells were infected with the resulting lentivirus at an MOI of 10 in the presence of 4 μg/ml polybrene overnight. The efficiency of infection was determined by detection of EGFP expression in response to heat shock (incubation at 43°C for 1h) using fluorescent microscopy. Between 70 and 90% of the cells were infected by virus and expressed GFP in response to heat shock. Bortezomib was from LC Laboratories (Woburn, MA). The drugs puromycin, emetine, and nutlin-3a were from Sigma-Aldrich (St. Luis, MO).

### Western immunoblotting

Total protein extracts from cells were prepared in RIPA buffer (150 mM NaCl, 1% SDS, 10 mM Tris (pH 8.0), 1% sodium deoxycholate, 1% NP-40) containing a protease inhibitor cocktail (Sigma, St. Louis, MO). 10 μg of the protein extracts were separated by electrophoresis in 4-20% gradient polyacrylamide gels with SDS and then transferred to nylon PVDF membranes (Amersham, Piscataway, NJ). Membranes were pre-incubated overnight in 5% milk solution, and then incubated for 1 h in 1% milk solution with primary antibodies at 1:1000 dilutions. The following primary antibodies were used: rabbit anti-Hsp70A1 inducible form (Assay Designs/StressGen, Victoria, BC, Canada) and rabbit anti-ubiquitin (Santa Cruz Biotechnology, Santa Cruz, CA). Rabbit anti-tubulin antibody was used to control for protein loading (Santa Cruz Biotechnology, Santa Cruz, CA). Apoptosis was analyzed by detection of caspase-specific cleavage of PARP (antibody from Santa Cruz Biotechnology, Santa Cruz, CA). HRP-conjugated secondary anti-rabbit antibodies were purchased from Santa Cruz Biotechnology. ECL detection reagent was from Perkin Elmer (Shelton, CT).

### Northern blotting and hybridization

Total RNA (10 μg) from HeLa, HT1080, or HCT116 cells was analyzed by Northern blot hybridization with probes specific to the Hsp70A1 and GAPDH genes. The Hsp70A1 probe consisted of a PCR fragment that was generated from the Hsp70A1 cDNA (Invitrogen, Carlsbad, CA) with primers specific for the Hsp70A1 coding sequence (A1s: 5' CCA CCA TCC CCA CCA AGC AGA C3'; A1a: 5' CAT GAA CCA TCC TCT CCA CCT 3'). All other hybridization probes were generated by restriction endonuclease digestion from cDNA clones (ORIGENE).

### In vivo ^35^S-protein labeling

HeLa cells were treated with drugs and their combinations according to the protocols of experiments. The regular culture medium was changed to a methionine/cysteine-free medium supplemented with ^35^S-methionine and ^35^S-cysteine (50 μCi/ml) (New England Nuclear/Perkin Elmer, Shelton, CT) and the cells were incubated for an additional 60 min in the presence of drugs. Cytoplasmic protein extracts were prepared according to the Dignam protocol [53]. Labeled proteins were analyzed by electrophoresis in 10% polyacrylamide gels followed by autoradiography.

### Cell viability assay

A cell viability assay was used to determine the cytotoxicity of drugs and drug combinations. Cells were grown to 75-80% confluency and then treated for 16 h with various combinations and doses of bortezomib and puromycin, or hyperthermia (43°C for 1h) and bortezomib. Cells were collected by trypsinization and a 1:50 dilution was seeded in fresh medium without any drugs in a 12-well plate. Cell viability was determined 72 h later by methylene blue staining after fixation of the cells with 10% formaldehyde. Methylene blue was extracted by 0.1 M HCl and its absorbance was measured at 560 nm. All assays were done in duplicate and the graphs show the average data. Trypan blue assay was used to estimate MPC11 MM cell viability [54].

### In vivo assay for general toxicity of drug treatments

To evaluate general toxicity, 5 groups of 8 week old BALB/c mice (3 mice/group) were given drug injections (intraperitonealy) every two days (5 total injections). Group 1 was injected with 1 mg/kg bortezomib; group 2 received 5 mg/kg puromycin; group 3 received 0.5 mg/kg bortezomib; group 4 received 0.5 mg/kg bortezomib + 5 mg/kg puromycin; and group 5 was injected with PBS. Individual mouse body weights were determined daily and used to calculate the average weight for each group.

### In vivo assay for tumor growth in mice

MPC11 cells were washed and resuspended in sterile PBS. Cells were injected sub-cutaneous into two sites of the shaved abdominal area of BALB/c mice (2x10^5^ cells in a single injection). Tumors were measured in two dimensions using digital calipers and tumor volume was calculated as: tumor volume (mm^3^) = 0.5 tumor length x (tumor width)^2^. On day 8 post-injection, tumors were typically ~50 mm^3^ in size. Tumor-bearing mice were divided into four groups of five animals each and were given intraperitonealy drug injections every 2-3 days beginning on day 9. The control group (Group 1) received PBS injections. Group 2 was injected with 5 mg/kg puromycin in PBS. Group 3 received 0.5 mg/kg bortezomib in PBS. Group 4 was injected with a combination of the indicated doses of bortezomib and puromycin. Tumors were measured every 2 days after the first drug injection. Animals were euthanized if tumors grew to > 1200 mm3.
